# Shifting reasons for older men remaining uncircumcised: Findings from a pre- and post-demand creation intervention among men aged 25–39 years in western Kenya

**DOI:** 10.1371/journal.pgph.0003188

**Published:** 2024-05-31

**Authors:** Kawango Agot, Jacob Onyango, George Otieno, Paul Musingila, Susan Gachau, Marylyn Ochillo, Jonathan Grund, Rachael Joseph, Edward Mboya, Spala Ohaga, Dickens Omondi, Elijah Odoyo-June

**Affiliations:** 1 Impact Research and Development Organization, Kisumu, Kenya; 2 Center for Microbiology Research, Kenya Medical Research Institute, Kisumu, Kenya; 3 Division of Global HIV and TB (DGHT), Center for Global Health (CGH), US Centers for Disease Control and Prevention, Kisumu, Kenya; 4 Division of Global HIV and TB (DGHT), Center for Global Health (CGH), US Centers for Disease Control and Prevention, Pretoria, South Africa; 5 Jaramogi Oginga Odinga University of Science and Technology, Bondo, Kenya; Makerere University, UGANDA

## Abstract

Voluntary medical male circumcision (VMMC) reduces men’s risk of acquiring Human immunodeficiency virus (HIV) through vaginal sex. However, VMMC uptake remains lowest among Kenyan men ages 25–39 years among whom the impact on reducing population-level HIV incidence was estimated to be greatest at the start of the study in 2014. We conducted a pre- and post-intervention survey as part of a cluster randomized controlled trial to determine the effect of two interventions (interpersonal communication (IPC) and dedicated service outlets (DSO), delivered individually or together) on improving VMMC uptake among men ages 25–39 years in western Kenya between 2014 and 2016. The study had three intervention arms and a control arm. In arm one, an IPC toolkit was used to address barriers to VMMC. In arm two, men were referred to DSO that were modified to address their preferences. Arm three combined the IPC and DSO. The control arm had standard of care. At baseline, uncircumcised men ranked the top three reasons for remaining uncircumcised. An IPC demand creation toolkit was used to address the identified barriers and men were referred for VMMC at study-designated facilities. At follow-up, those who remained uncircumcised were again asked to rank the top three reasons for not getting circumcised. There was inconsistency in ranking of reported barriers at pre- and post- intervention: ‘time/venue not convenient’ was ranked third at baseline and seventh at follow-up; ‘too busy to go for circumcision’ was tenth at baseline but second at follow-up, and concern about ‘what I/family will eat’ was ranked first at both baseline and follow-up, but the proportion reduced from 62% to 28%. Men ages 25–39 years cited a variety of logistical and psychosocial barriers to receiving VMMC. After exposure to IPC, most of these barriers shifted while some remained the same. Additional innovative interventions to address on-going and shifting barriers may help improve VMMC uptake among older men.

## Introduction

Three randomized controlled trials conducted in Kenya, South Africa, and Uganda showed that VMMC reduces the rate of heterosexual men acquiring HIV through vaginal sex by about 60% [1–3]. In 2007 the World Health Organization (WHO) and the Joint United Nations Programme on HIV/AIDS (UNAIDS) recommended countries with a high HIV prevalence and low rates of male circumcision to consider scaling-up VMMC as part of a comprehensive HIV prevention package [4]. Kenya’s VMMC program initially focused on Nyanza Province of western Kenya, a region with the highest HIV prevalence in the country at the start of the study—15.1% against 5.6% nationally [5]—and predominately traditionally non-circumcising *Luo* ethnic community [6–8].

Globally, between 2008 and 2022, about 35 million men and boys had accessed VMMC services across 15 priority countries in sub-Saharan African (SSA) and this has contributed to averting new infections and related healthcare costs [9]. In Kenya, a total of 2,143,017 VMMC were done in Kenya between 2008 and 2020, with 689,761 VMMC being done between 2017 and 2020 [9]. VMMC is reported to have already averted a substantial number of HIV infections. Modelling estimate that the nearly 23 million circumcisions performed by the end of 2018 in 15 priority countries in East and Southern Africa prevented approximately 250,000 new HIV infections and it was projected that the number of infections averted by VMMC would increase to 1.5 million by 2030 [10]. In Kenya, the United States Agency for International Development (USAID) estimated that reaching 60% of the target population by 2014 could avert 47,000 infections by 2025 [11] while another report projected that VMMCs conducted through 2015 would avert 21,000–33,000 infections through 2030 [12]. However, research has also shown that VMMC, even when highly cost-effective as an HIV intervention, is more difficult to advocate for than other direct interventions such as pre-exposure prophylaxis (PrEP) [13]. On the other hand, the scale-up of antiretroviral therapy (ART) programs and prevention interventions such as PrEP has resulted in a decline in HIV incidence in several countries [14–16]. A mathematical modelling study showed that if both VMMC and PrEP were scaled up to 80% coverage, VMMC, with an assumed efficacy of 60%, had a lower impact than oral PrEP, with an assumed effectiveness of 71% [17].

Despite successes in rolling out the VMMC program [6, 10, 18], the uptake in Kenya remain lowest among older men [19–22]. The Kenya Population–based HIV Impact Assessment Survey report showed that VMMC prevalence among males aged ≥ 20 years in the priority counties was 46.9% compared to 63.2% among males aged 15–19 years [23] while the U.S. President’s Emergency Plan for AIDS Relief (PEPFAR) report showed only 5% of the circumcisions performed from 2015–2021 were among men ages ≥25 years [24], indicating that reaching older men with VMMC services remains an ongoing challenge.

The lower uptake of VMMC among older men has been attributed to various barriers. Studies have shown that generally men find it difficult to seek health care [25–28]. In the case of VMMC, this is compounded by the fact that it requires healthy men to undergo a surgical procedure involving considerable discomfort and inconvenience, and provides only partial protection against an uncertain individual risk of acquiring HIV [4, 29]. A number of approaches have been implemented to increase demand for VMMC in Eastern and Southern Africa, including: providing conditional economic compensation [30, 31], providing fixed compensation and lottery-based rewards [32, 33], demand generation by age prioritization and outreach services [34, 35], information and motivation through pregnant female partners [36], and peer referral incentive [37]. At best, these interventions have resulted in modest increase in the uptake of circumcision by men above 20 years of age.

Individual-level barriers have been highlighted in various countries where VMMC programs have been implemented [38]. The fear of pain and potential complications [7, 20, 39], discomfort of sharing waiting areas with younger clients [40], HIV testing [39], the perception that circumcision reduces penis sensitivity thus sexual pleasure [41], difficulty in adhering to sexual abstinence for 6 weeks post-operation [42], loss of income during the healing period [7, 20, 43, 44] and the concern that male circumcision is against cultural and ethnic identity [2, 19, 42], have been documented as the key barriers to VMMC uptake among adult men in traditionally non-circumcising communities.

We implemented a pre- and post- intervention cross-sectional survey between 2014 and 2016 as part of a cluster randomized controlled trial (cRCT) called the Target, Speed and Coverage (TASCO) study in four counties of former Nyanza Province. The interventions included IPC to address individualized barriers, and DSO to prioritize older men seeking VMMC services. The results of the study did not show that these strategies were effective in increasing VMMC uptake among older men [45]. In this manuscript we analyzed the agreement between reported barriers to circumcision in the pre- and post-intervention periods to explore if older men may be shifting their reasons for remaining uncircumcised. We posited that if men selected the same barriers at pre- and post-intervention, then the intervention was likely not effective in making them make a decision to get circumcised, but if men selected different reasons for remaining uncircumcised post-intervention, then they have likely decided against being circumcised whether or not their barriers are addressed, or that there are other barriers that were not highly ranked at enrollment but which were also important enough to deter them from getting circumcised.

## Methods

As part of the formative phase of the TASCO study, we conducted a systematic review of over 80 articles and research studies conducted in different countries of SSA on barriers and facilitators to VMMC uptake. Second, we conducted a formative qualitative study (focus group discussions (FGDs) and in-depth interviews (IDIs)) and obtained data about perceptions and impressions of circumcised men and their partners, and uncircumcised men and their partners, about barriers and facilitators to VMMC specifically among men aged 25–39 years in the Nyanza region where the study took place. Data from FGDs and IDIs were analyzed thematically to extract information on the VMMC primary barriers and facilitators. To ensure the list was exhaustive, we triangulated barriers from the systematic review, FGDs and IDIs to generate a list of 28 barriers (in order of most cited in literature and during the formative phase). Third, the lead author who has extensive experience in VMMC research and service delivery and other authors who were engaged in VMMC programs in Nyanza (JO, SO and DO) drafted the toolkit (*Interpersonal Communication*: *Toolkit for Addressing Barriers and Facilitators to Voluntary Medical Male Circumcision for Older Men in Nyanza Region*, *Kenya* (S1 File) based on data from the two sources.

A 3-day workshop was then convened by the 4 experts that also included a member of the Luo Council of Elders, a media person, a circumcision surgeon, a VMMC counsellor, and a community advocate to review the draft toolkit, paying particular attention to what should be included in the talking points on how to respond to the barriers and reinforce the facilitators, and what possible myths and misconceptions may arise and how to address them. Male lay counselors served as research assistants (RAs) and were trained on how to administer the toolkit. The toolkit provided the following instructions: *Begin the intervention by asking the participant why he has not gone to be circumcised; write all of the barriers mentioned by the participant to guide the discussion; using talking points in the sections of the Toolkit related to each barrier*, *address each reported barrier carefully and completely*, *always ensuring the person is engaged in the discussion; the goal of this conversation is to provide correct and complete information in a relaxed and conversational manner; revisit each stated barrier*, *exploring the participant’s beliefs and thoughts after discussion of each; after the discussion*, *explore whether the same barriers would still deter him from getting circumcised; address any new or lingering concerns*. *After addressing all of the questions and concerns*, *finish the conversation and encourage the participant to seek VMMC at a designated facility*.

For each of the cited barriers, the Toolkit contained talking points to guide the discussion. For example, an excerpt of contents in talking points provided for those who said ‘VMMC is against my culture’ as a barrier were: *Explore what he means by culture loss*, *correct any misconceptions*, *then say*: “VMMC means Voluntary Medical Male Circumcision; as the name suggests, this is done purely for MEDICAL reasons and is voluntary; VMMC is not done for cultural reasons; it is not based on any season or accompanied by any ritual; it is not done to make one identify with a community; unlike traditional male circumcision (MC) for cultural reasons, VMMC is not done as a rite of passage to adulthood; thus, VMMC is a medical procedure that has nothing to do with cultural beliefs; VMMC is purely for HIV prevention and other medical benefits such as preventing certain sexually transmitted infections (STIs), reducing the risk of penile cancer in men and cervical cancer in women; VMMC also makes it easier to clean the genital area and to wear a condom; VMMC is performed by trained health care providers while cultural MC is done by traditional circumcisers; a large number of men your age have already been circumcised and are satisfied with their decision to get circumcised.” *(Correct any additional misconceptions mentioned by the client that are not covered in the talking points above*.*)*

The study had four arms implemented in geographic clusters: IPC-only, DSO-only, IPC+DSO combined, and control [45]. Participant recruitment was conducted between February 16, 2015 to August 02, 2015. In summary, at baseline uncircumcised participants were asked to list all the reasons for not being circumcised, then rank the top three in order of importance (1 = most important). The RAs used the IPC Toolkit to address the barriers cited and correct any misinformation. Participants in the IPC-only clusters received a single-session IPC intervention addressing their concerns, fears and misconceptions and were referred for VMMC in health facilities with no preferential services for older males. Examples of the barriers discussed included pain, circumcision being against culture or religion, complications, period of sexual abstinence, subsistence during healing period, provider’s gender, age mixing at facility, and effect on sexual libido, among other topics (S1 File).

In the IPC+DSO clusters, participants received IPC and were referred to designated health facilities where older men were fast tracked to reduce waiting time, served in a separate waiting bay dedicated to men aged ≥25 years, served by male providers only, and offered flexible services including on designated weekends and evenings. A follow-up survey was conducted among participants in the IPC-only and IPC+DSO clusters from 4–7 months after baseline, when follow-up period for all participants had ended. After receiving the IPC intervention, participants in both arms were given a referral coupon to present when seeking VMMC services; facility-based RAs asked men seeking VMMC during the study period if they were participating in the TASCO study, and had a referral coupon. The identity of those who reported being a study participant but did not have a coupon was verified by cross-checking responses to selected baseline questions. Service uptake was defined as having received VMMC at any of the designated study facilities within three months of enrollment. At post-intervention, participants who were not circumcised at baseline were contacted and asked if they had gone for VMMC; those reporting having been circumcised were confirmed through physical verification. Participants who did not go for VMMC were asked to list all the reasons for not going and to rank the top three reasons in order of importance. Data were entered in a password-protected Microsoft Access 2007 database then imported to STATA version 16.1 for analysis.

### Data analysis

We summarized the categorical variables for pre- and post-intervention data using frequency counts and percentages. The reported barriers were stratified and analyzed by the two study arms at baseline and follow-up. A Multicriteria Decision Making (MCDM) [46] approach was adopted to generate hierarchy weights for each of the top three ranked barriers to enable issue aggregation. MCDM is useful for screening, prioritizing, ranking or selecting the alternatives based on human judgment from among a finite set of alternatives. The MCDM models allocate hierarchy weights which provide information about the relative importance of the considered items. A pair-wise comparative analysis (baseline and follow-up) was conducted to establish the priority issue or foremost barrier. Roszkowska and colleagues [47] proposed an approximation for criteria weights based on rank-order centroid weight method and provided different formulas for different *n* values when *n* represents the number of variables being ranked. In the case for TASCO with 3 ranking categories the weights were generated using Rank Order Centroid [48];

$$W_{k}(ROC)=\frac{1}{n}\sum_{j=k}^{n}\frac{1}{j}$$

where k is the rank of the kth criterion, k = 1, 2,…, n.; w_1_ = 0.61; w_2_ = 0.28; w_3_ = 0.11. w_1_ was the weight applied to all variables ranked 1, w_2_ for rank 2 and w_3_ for rank 3 while j = 1,2, and 3, representing first, second and third respectively. Sum of weights for the barriers at baseline and follow-up were then calculated as a linear combination of ranks 1, 2 and 3 using procedures described by Buede and Maxwell [49]. This weighting allowed ranking of barriers at baseline and follow-up from the most important to the least important.

The barriers were then re-coded to differentiate those from baseline and follow-up. Each barrier at baseline and follow-up was then re-coded to store three ranks (1, 2 and 3). This resulted in a three by three (3 x 3) comparison matrix between baseline and follow-up ranking of the same barrier. This also resulted in several possible outcomes for each barrier, for example, there were participants who ranked a barrier position 1 at baseline and still ranked the same at position 1 at follow-up, some position 2 and retained it at position 2, while there are those who ranked the same at position 3 and also retained it at position 3 at follow-up.

When a baseline barrier was cross-tabulated with a follow-up barrier, it formed a 3x3 matrix where the leading diagonal of (rank 1 and 1) or (2 and 2) or (3 and 3) was considered ‘perfect agreement’. The other combinations were considered some agreement or misclassified. A new binary variable was then created and coded as 1 for some agreement /perfect agreement since there was only one perfect agreement and 0 otherwise. Frequency and percent of those ‘perfect agreements/some agreement’ was calculated for each of the 28 barriers and considered as an outcome variable.

Pearson’s Chi-square adjusted for clustering was used to assess the association between agreement pre- and post-intervention and categorical variables. Cohen’s kappa statistic, κ, was used to assess agreement between barriers to circumcision pre- and post-intervention [50].

To account for clustering in the study design, we employed generalized linear mixed effect modelling framework and logistic regression, and we determined predictors of agreement between barriers to circumcision pre- and post-intervention among categories of explanatory variables using bivariate and multivariable logistic model. A p-value of ≤ 0,05 was considered significant. The analysis was completed using STATA version 16.1 (STATA Corporation, College Station, Texas, USA).

### Ethical considerations

Ethical approvals were obtained from the Kenyatta National Hospital/University of Nairobi Ethics Review Committee (P36/02/2013). All participants provided written informed consent prior to taking part in the study activities. The study was also reviewed in accordance with the U.S. Centers for Disease Control and Prevention (CDC) human research protection procedures and was determined to be research, but the CDC investigators did not interact with human subjects or have access to identifiable data or specimens for research purposes.

## Results

**Fig 1:**
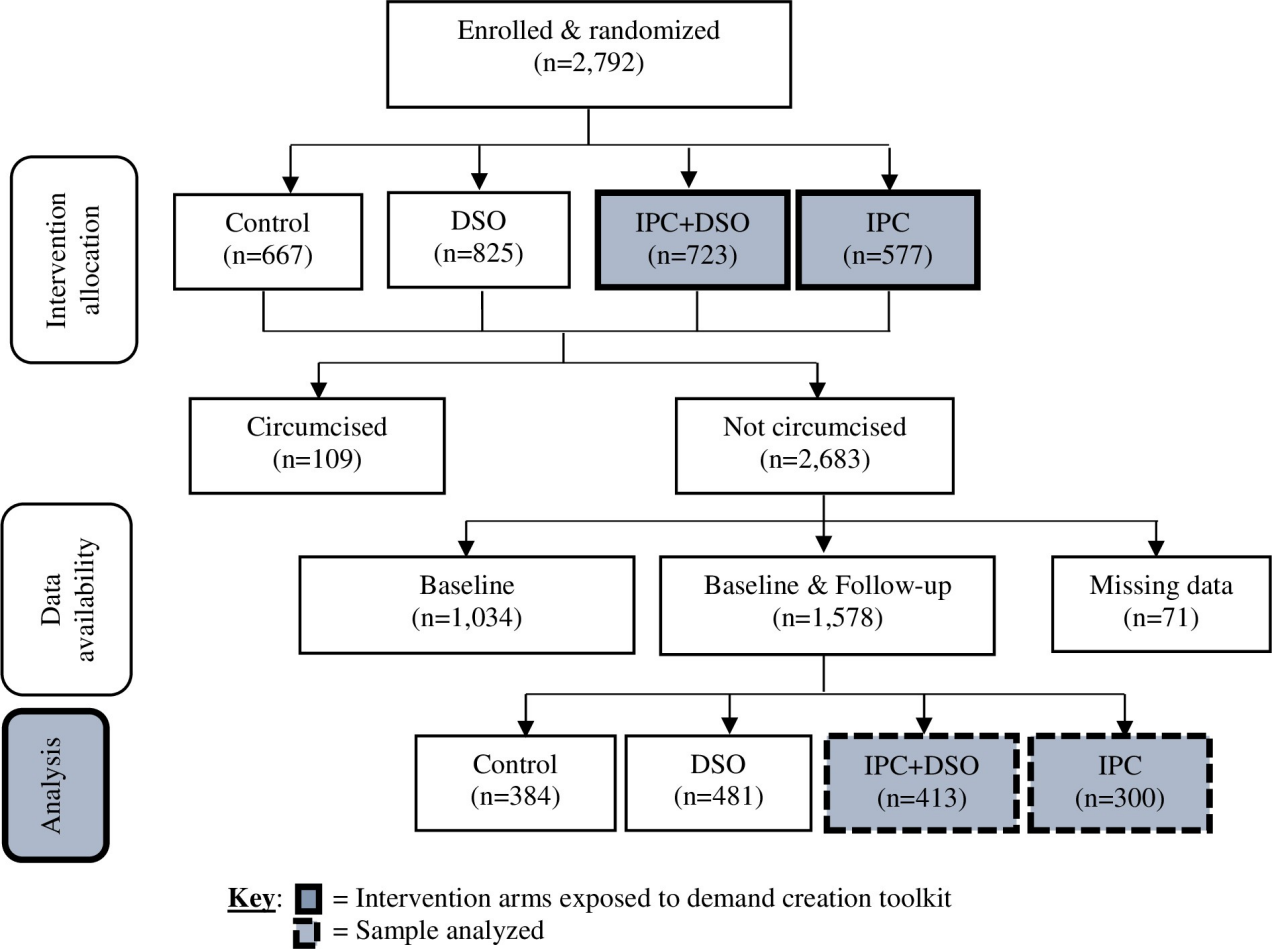
Flowchart of participant enrollment and follow-up, TASCO study.

Of the 2,683 males who were uncircumcised at the start of the study and remained uncircumcised at the end, 1,649 were interviewed and their data at baseline and follow-up included in this analysis. Follow-up rates were uniformly distributed across the study arm except IPC which had slightly lower rate compared to the rest: control (59%), DSO (61%), DSO+IPC (60%), and IPC (54%). We further restricted the sample for the analysis to only those randomized to IPC-only and IPC+DSO clusters (n = 713) to whom the demand creation tool kit was administered as shown in **Fig 1**.

### Reported barriers to VMMC uptake

**Table 1** shows barriers to VMMC uptake among uncircumcised men aged 25–39 years at baseline and follow-up. At baseline, the top three barriers to circumcision were: i) what I/family will eat, ii) pain or fear of complications, and iii) time/venue not convenient. At follow-up, the top three barriers to circumcision were: i) what I/family will eat, ii) too busy to go for circumcision, and iii) pain or fear of complications. Whereas ‘time/venue not convenient’ dropped in ranking from third at baseline to seventh at follow-up, ‘too busy to go for circumcision’ was ranked tenth at baseline but moved to second position at follow-up. Of the 713 participants, 261 (63.2%) reported ‘what I/family will eat’ as a barrier at baseline for those in IPC+DSO arm, whereas at follow-up, only 114 (28.0%) mentioned it as a barrier (p-value = 0.012). The proportion citing ‘pain or fear of complication’ at baseline and follow-up was 281 (68.0%) and 43 (10.4%), respectively, p-value = 0.34. Only nine (2.2%) mentioned ‘too busy to go for circumcision’ at baseline while 118 (28.6%) cited it at follow-up (p-value = 0.675).

**Table 1:** Reported barriers to VMMC uptake among uncircumcised men at baseline and at follow-up (N = 713), TASCO study.

			Baseline					Follow-up				
				DSO+IPC	IPC				DSO+IPC		IPC	
	Sum	Rank	Total(N)	n(%)	n(%)	Sum	Rank	Total(N)	n(%)	p^a^	n(%)	p^a^
**What I/family will eat**	201	1				98	1			0.91		0.86
No			269(37.7)	152(36.8)	117(39.0)			521(73.1)	299(72.4)		222(74.0)	
Yes			444(62.3)	261(63.2)	183(61.0)			192(26.9)	114(27.6)		78(26.0)	
**Pain/fear of complications**	180	2				27	3			0.91		0.73
No			225(31.6)	132(32.0)	93(31.0)			633(88.8)	370(89.6)		263(87.7)	
Yes			488(68.4)	281(68.0)	207(69.0)			80(11.2)	43(10.4)		37(12.3)	
**Time/venue not convenient**	45	3				4	7			<0.01		0.55
No			566(79.4)	336(81.4)	230(76.7)			701(98.3)	407(98.5)		294(98.0)	
Yes			147(20.6)	77(18.6)	70(23.3)			12(1.7)	6(1.5)		6(2.0)	
**Contrary to religion**	29	4				4	7			<0.01		0.10
No			638(89.5)	369(89.3)	269(89.7)			700(98.2)	407(98.5)		293(97.7)	
Yes			75(10.5)	44(10.7)	31(10.3)			13(1.8)	6(1.5)		7(2.3)	
**Reduce sexual function**	21	5				4	7			0.76		0.36
No			651(91.3)	379(91.8)	272(90.7)			704(98.7)	411(99.5)		293(97.7)	
Yes			62(8.7)	34(8.2)	28(9.3)			9(1.3)	2(0.5)		7(2.3)	
**Waiting time too long**	21	5				4	7			0.01		0.02
No			639(89.6)	369(89.3)	270(90.0)			707(99.2)	410(99.3)		297(99.0)	
Yes			74(10.4)	44(10.7)	30(10.0)			6(0.8)	3(0.7)		3(1.0)	
**Sexual abstinence period long**	19	6				4	7			0.04		0.67
No			630(88.4)	380(92.0)	250(83.3)			708(99.3)	412(99.8)		296(98.7)	
Yes			83(11.6)	33(8.0)	50(16.7)			5(0.7)	1(0.2)		4(1.3)	
**VMMC is for younger people**	11	7				6	5			0.54		0.17
No			668(93.7)	384(93.0)	284(94.7)			700(98.2)	406(98.3)		294(98.0)	
Yes			45(6.3)	29(7.0)	16(5.3)			13(1.8)	7(1.7)		6(2.0)	
**Penis will shrink**	4	8				1	10			0.03		<0.01
No			699(98.0)	410(99.3)	289(96.3)			710(99.6)	413(100.0)		297(99.0)	
Yes			14(2.0)	3(0.7)	11(3.7)			3(0.4)	0(0.0)		3(1.0)	
**Female partner opposed**	4	8				0.7	11			0.02		0.01
No			697(97.8)	406(98.3)	291(97.0)			710(99.6)	413(100.0)		297(99.0)	
Yes			16(2.2)	7(1.7)	9(3.0)			3(0.4)	0(0.0)		3(1.0)	
**Increase sexual function**	3	9				0	12			0.34		0.01
No			699(98.0)	407(98.5)	292(97.3)			713(100.0)	413(100.0)		300(100.0)	
Yes			14(2.0)	6(1.5)	8(2.7)							
**Busy to go for circumcision**	1	10				50	2			0.67		0.24
No			700(98.2)	404(97.8)	296(98.7)			520(72.9)	295(71.4)		225(75.0)	
Yes			13(1.8)	9(2.2)	4(1.3)			193(27.1)	118(28.6)		75(25.0)	

^a^ Chi-square test of independence (baseline IPC vs. follow-up IPC; baseline DSO +IPC vs. follow-up DSO +IPC)

Of those in the IPC arm, at baseline 183 (61%) cited ‘what I/family will eat’ as the main concern while at follow-up, only 78 (26%) cited the same barrier. Similarly, at baseline, 4 (1.3%) of the participants in the IPC group reported ‘too busy to go for circumcision’ while those reporting the same increased to 75 (25.0%) at follow-up.

Kappa statistics shows the agreement between barriers to circumcision pre- and post-intervention in **Table 2**. The barriers for which *expected agreement* was not equal to *agreement (p-value* ≤ *0*.*05)*, meaning those in which we can reject the hypothesis that participants selected barriers at random were: contrary to religion, pain/fear of complications, what I/family will eat, time/venue not convenient, reduce sexual pleasure, not at risk, and served by female providers. Among those barriers with some level of agreement, the agreement observed was slight (kappa = 0.01–0.20); none was considered fair (kappa = 0.21–0.40), moderate (kappa = 0.41–0.60), substantial (kappa = 0.61–0.80) or almost perfect agreement (kappa = 0.81–1.00). Increased sexual function, lining up with young people, and being served by young health care providers were mentioned at baseline and not at follow-up.

**Table 2:** Level of agreement for each barrier at baseline and follow-up (N = 713), TASCO study.

Barrier	Baseline	Follow-up	Agreement (%)	Expected agreement (%)	Kappa	p-value
What I/family will eat	444(62.3)	192(26.9)	49.79	44.34	0.098	0.000
Pain/fear of complications	488(68.4)	80(11.2)	38.29	35.70	0.040	0.009
Time/venue not convenient	147(20.6)	12(1.7)	78.82	78.39	0.020	0.136
Contrary to religion	75(10.5)	13(1.8)	89.62	88.04	0.132	0.000
Reduce sexual function	62(8.7)	9(1.3)	91.44	90.26	0.122	0.000
Waiting time too long	74(10.4)	6(0.8)	89.06	88.95	0.010	0.306
Sexual abstinence period long	83(11.6)	5(0.7)	87.66	87.82	-0.013	0.792
VMMC is for younger people	45(6.3)	13(1.8)	92.71	92.60	0.077	0.056
Penis will shrink	14(2.0)	3(0.4)	97.62	97.63	-0.007	0.597
Family opposed	13(1.8)	2(0.3)	96.21	96.01	0.050	0.090
Busy to go for circumcision	13(1.8)	193(27.1)	72.23	72.10	0.005	0.381
I am not at risk	21(2.9)	10(1.4)	96.21	95.73	0.112	0.001
Served by female providers	22(3.1)	1(0.1)	97.05	96.78	0.085	0.000
Peer opposed	12(1.7)	1(0.1)	98.18	98.18	-0.003	0.552
Female partner opposed	16(2.2)	3(0.4)	97.34	97.35	-0.007	0.604
Lack of information	14(2.0)	1(0.1	97.90	97.90	-0.003	0.556

^**¥**^≤ 0 as indicating no agreement; 0.01–0.20 as none to slight; 0.21–0.40 as fair; 0.41–0.60 as moderate; 0.61–0.80 as substantial, and

0.81–1.00 as almost perfect agreement

Frequency & percentages represent only those who chose ‘yes’ to the barriers at baseline as well as at follow-up

### Agreement between barriers to circumcision pre- and post-intervention

Most participants were from Kisumu County (325, 45.6%) and the majority reported being married (615, 86.3%), with only 77 (11.0%) reporting being single (**Table 3**). A larger proportion of the participants (280, 39.3%) were less than 30 years of age compared to age groups 30–34 (34%) and 35–39 (27%); nearly all 708 (99.3%) were Christians, and more than two-thirds (494, 69.3%) reported primary level of education. Participants who were single were more likely to inconsistently report barriers compared to those who were married, with nearing significance (92.0% vs. 82.0%, p = 0.055). Among those with a college/university level of education (n = 67), only 5% consistently reported barriers compared to those in secondary school (5% vs. 0.15%, p = 0.019).

**Table 3:** Agreement between barriers to circumcision pre- and post- intervention (n = 713), TASCO study.

	Consistent		Inconsistent	
	n (%)	n (%)	n (%)	P-value^¥^
**Intervention group**				0.515
IPC	300(42.1)	44(14.7)	256(85.3)	
DSO+IPC	413(57.9)	68(16.5)	345(83.5)	
**Age in years**				0.492
25–29	280(39.3)	45(16.1)	235(83.9)	
30–34	243(34.1)	42(17.3)	201(82.7)	
35–39	190(26.6)	25(13.2)	165(86.8)	
**County**				0.154
Homabay	95(13.3)	20(21.1)	75(78.9)	
Kisumu	325(45.6)	48(14.8)	277(85.2)	
Migori	135(18.9)	15(11.1)	120(88.9)	
Siaya	158(22.2)	29(18.4)	129(81.6)	
**Marital status** *				0.055
Divorced/Separated/Widowed	21(2.9)	2(9.5)	19(90.5)	
Married	615(86.3)	104(16.9)	511(83.1)	
Single	77(10.8)	6(7.8)	71(92.2)	
**Religion**				0.791
Christian	708(99.3)	111(15.7)	597(84.3)	
Not Christians	5(0.7)	1(20.0)	4(80.0)	
**Education level**				**0.019**
Up to Primary	494(69.3)	87(17.6)	407(82.4)	
Secondary	152(21.3)	22(14.5)	130(85.5)	
College/University	67(9.4)	3(4.5)	64(95.5)	
**Employment Status**				0.493
Salaried/Wages	403(56.5)	60(14.9)	343(85.1)	
Unemployed	310(43.5)	52(16.8)	258(83.2)	

¥Chi-square/Fisher’s exact p value adjusted for cluster

*Borderline significance

**Table 4** reports unadjusted and adjusted analysis of factors associated with agreement between barriers to circumcision pre- and post-intervention. After adjusting for other variables, only county of residence and education level independently predicted agreement between barriers pre- and post-intervention. Agreement between the two time periods tended to increase among participants with secondary education (OR = 2.84, 95% CI 1.07–7.77, p = 0.036); however, those in the IPC+DSO arm were 74% less likely to report the same barriers pre- and post-intervention compared to those in the IPC-only arm (OR = 0.26, 95% CI 0.08–0.86, p = 0.028). Participants from Migori County were over two and a half times more likely to report the same barriers pre- and post- intervention compared to those from Homabay County (OR = 2.56, 95% CI 1.20–5.35, p = 0.014).

**Table 4:** Predictors of agreement between barriers to circumcision pre- and post-intervention.

		Unadjusted OR		Adjusted OR	
Variable/Factor	Total (N%)	OR (95% CI)	p value	OR (95% CI)	p value
**Treatment group**					
IPC	300(42.1)	Ref			
DSO+IPC	413(57.9)	0.94 (0.53–1.68)	0.842	0.98 (0.60–1.61)	0.957
**County**					
Homabay	95(13.3)	Ref			
Kisumu	325(45.6)	1.62 (0.76–3.49)	0.213	1.5 (0.85–2.94)	0.145
Migori	135(18.9)	2.21 (0.97–5.03)	0.060	2.5 (1.20–5.35)	**0.014**
Siaya	158(22.2)	1.22 (0.59–2.54)	0.588	1.2 (0.62–2.34)	0.576
**Age group in years**					
25–29	280(39.3)	Ref			
30–34	243(34.1)	0.86 (0.54–1.39)	0.547		
35–39	190(26.6)	1.2 (0.70–2.05)	0.513		
**Marital status**					
Married	615(86.3)	Ref			
Single	77(10.8)	1.35 (0.25–7.30)	0.040	1.12 (0.20–6.21)	0.809
Divorced/Separated/Widowed	21(2.9)	0.54 (0.12–2.40)	0.421	0.49 (0.11–2.18)	0.354
**Employment Status**					
Salaries/Wages	403(56.5)	Ref			
Unemployed	310(43.5)	0.9 (0.60–1.36)	0.611		
**Education**					
Up to Primary	494(69.3)	Ref			
Secondary	152(21.3)	1.23 (0.73–2.05)	0.435	2.89 (1.07–7.77)	**0.036**
College/University	67(9.4)	4.37 (1.33–14.31)	0.015	1.98 (0.56–7.00)	0.284
**Interaction (Treat#Education level)**					
Dso+Ipc#Secondary				0.26 (0.08–0.86)	**0.028**

## Discussion

The TASCO study sought to assess the impact of two interventions–IPC and DSO–delivered individually or in combination, on the uptake of VMMC among men ages 25–39 years living in western Kenya. Studies have reported that interventions that use a blend of promotional, interpersonal communications, and modified service delivery that is more accessible generally have positive effects than those that use only routine service delivery strategies [51]. However, increasing VMMC uptake among older men at greatest risk of HIV infection has remained a challenge [8, 32, 35, 52, 53]. Given that older men experience social, cultural and medical barriers to accessing VMMC services [29], evidence suggests that IPC could play a stronger role in their decision to undergo VMMC [54]. IPC can facilitate a man’s transition from intention to getting circumcised to actual uptake of the services [55].

However, our results did not provide sufficient evidence that these strategies were effective in increasing VMMC uptake among older men; they might have addressed certain aspects of the barriers but not all [45], or participants may have changed their reasons for staying uncircumcised despite their concerns about and fears of circumcision being addressed through IPC and DSO interventions. Our findings on the effectiveness of IPC in addressing barriers to VMMC are comparable to the findings of a study that was conducted in Zimbabwe among men aged 20–35 years in 2018 to assess the impact of IPC approach. In this study, IPC demand creation was augmented by a Human- Centered Design (HCD)-informed approach. IPC agents conducted one-on-one sessions with uncircumcised clients using three tools: a segmentation typing tool, segment-specific targeted messaging and a pain-o-metre (visual aid) to guide discussions related to barriers. The study showed that the IPC demand creation approach plus HCD-informed, did not increase demand for VMMC among this population [56], similar to what our study found.

Our study explored the possibility that older men may have decided against being circumcised and were giving different or random reasons at different time points. In ranking individual barriers to VMMC at baseline and at follow-up, most participants who ranked a given barrier as first, second or third, at baseline did not rank the same barrier at the same position at follow-up. For example, the concern about ‘what I/family will eat’ was ranked first by 63.2% of participants at baseline and still ranked first at follow-up, but by 27.6% only. ‘Time/venue not convenient’ was ranked third at baseline and seventh at follow-up whereas ‘too busy to go for circumcision’ was ranked tenth at baseline but second at follow-up. Penis will shrink, sexual abstinence period long, peer opposed, female partner opposed, and lack of information showed negative Kappa statistics which implied no agreement pre-and post-intervention in terms of the barriers to VMMC.

There are several ways to interpret these inconsistencies. One, participants with agreement may have found that the interventions provided did not effectively address their primary concerns hence cited the same barriers at follow-up; two, those with no or slight agreement may have had their top three concerns addressed by the interventions but other concerns previously ranked lower remained significant enough to preclude seeking out VMMC; and three, participants could have made a decision to never get circumcised and gave arbitrary reasons which they could not recall when asked again within a span of just 4–7 months between baseline and follow-up. The finding of shifting reasons for remaining uncircumcised among men may indicate the role of other important but tacit barriers to VMMC uptake and that other demand creation interventions may be required, but could also mean older men may have just decided they would not get circumcised and no intervention would change their minds [30–37].

Economic barriers such as concern about opportunity costs for time lost from work (concerned about ‘what I/family will eat”) and the cost of travel may prevent men who are economically disadvantaged from accessing health services [57]. Recent literature reviews have considered that carefully selected economic interventions may be a useful targeted strategy to enhance VMMC coverage in SSA among older men and may be a way of reducing inequity in accessing VMMC [38, 57–59]. Economic compensation have been explored for a variety of HIV-related behaviors [60, 61], HIV testing [60, 62], and linkage to HIV treatment and adherence to ART [59, 60, 62–64]. However, there have been concerns about the sustainability of this strategy in the long term. Many countries have been concerned about incorporating incentives into their health systems, worrying that they may distort health care delivery, and pitting interventions against each other [31].

VMMC uptake in 2020 and 2021 in Kenya was very minimal compared to the previous years and this may partly be attributed to reduction in VMMC funding globally [65] following the transition from rapid to sustainability phase in addition to the individualized barriers to VMMC which have remained the same. Recent literature indicate that these barriers still have some implication for VMMC demand-creation given that a number of innovative demand creation approaches that have been implemented in various set-ups to address them have not resulted in significantly higher VMMC uptake. Specifically, a study conducted in 2019 to investigate the attitudes and key challenges to VMMC adoption found that panic, perceived surgical complications, cost in accessing VMMC services, and involvement of female health workers in the circumcision team, were some of the barriers to VMMC uptake among men in Malawi [66]. Tusabe, et al., [67] in a study conducted in 2020, found that fear of pain and compulsory HIV testing, long healing time, financial constraints, perceived interruption of God’s plan, loss of male fertility and involvement of female health workers were barriers to VMMC. Another study also conducted in 2020 in Namibia, found that myths and misconceptions attached to VMMC, age limitations, fear of pain and stigma associated with HIV, and long distances from health facilities negatively impacted VMMC uptake [68].

The study data also showed that education level independently predicted agreement between barriers to circumcision at pre- and post-intervention. Participants who had secondary school education and above showed higher likelihood of inconsistency compared to those who had attained primary or lower level education. It has been hypothesized that increased education would positively relate to stages of change in decision-making to improve one’s health [69] and that men with more education and knowledge are more ready to undergo VMMC [70]. However, the influence of education on circumcision decisions has showed varied results across countries. In Uganda, Tanzania, Kenya, and Ethiopia, circumcision prevalence was reported to be higher among individuals with secondary school and tertiary education while in Malawi and Lesotho, VMMC uptake has been reported to be lower among those with higher education [4]. These findings seem to strengthen the assertion by Mhagama and colleagues [71], that the level of education plays opposite roles in facilitating and deterring VMMC uptake. Further research to identify how education level may interact with other determinants to modify uptake of VMMC by older men is needed.

Our data show that most participants were married, Christians and inconsistently reported reasons for not being circumcised. The assumption is that uncircumcised older men who are married would not want to get circumcised because that would suggest to others (especially their female sexual partners) their intention to engage in extramarital sex and needed to protect themselves by getting circumcised. Another assumption is that those who are married no longer feel at risk of HIV acquisition [72]. Studies have reported a misconception that male circumcision increases promiscuity [21, 73–75] and that promoting VMMC may lead to more sexual partners that could, in turn, increase the HIV risk of circumcised men [76].

Additionally, it could be possible that the men saw no reason to get circumcised since their religion advocates for faithfulness. However, the influence of value systems associated with religious beliefs on the decision to get circumcised is debatable. Religion has been reported as a barrier to seeking VMMC in some studies of the acceptability of VMMC [77, 78]. In a study conducted in Nyanza region, Kenya, the fourth-ranked reason reported by men who did not choose VMMC was religion [79]. In another study in the Westonaria district of South Africa, about a third of the uncircumcised men reported that the reason they had not been circumcised is because circumcision is forbidden in their religion [80]. The association between VMMC and Christianity is understudied. In a study conducted in Iringa, Tanzania, a few years after VMMC was launched in the country, religion was not reported as a barrier to seeking MC [81]. However, given that nearly all participants in our study were Christians, we may not know if religion might have influenced their disinterest in VMMC. We recommend further in-depth research into the association between Christianity and VMMC.

This study had some limitations. The analysis looked at proportions of participants who reported a barrier at baseline and follow-up rather than what an individual said at baseline and follow-up; future studies may adopt data tools designed to track barriers mentioned at baseline and follow-up by each individual. Secondly, the response rate at follow-up was low compared to the baseline, which could have reduced the power to detect important changes. Furthermore, robust multivariate data analysis methods such as principal component analysis were not considered in this study even though the participants listed multiple reasons for not being circumcised at baseline and at follow-up. This is because the data set available for analysis was restricted to the top three reasons for remaining uncircumcised and excluded all the other reasons cited.

Despite these limitations, to our knowledge, this is the first study that attempted to look at consistency in stated reasons for remaining uncircumcised despite one’s top barriers being addressed real time. While previous studies have intervened on the known barriers and observed the effect on circumcision uptake, we went beyond that to address only barriers cited by participants and confirmed if those who remained uncircumcised were held back by the same barriers or if new barriers emerged. The emergence of new barriers and marked reduction in the proportion citing previous barriers in our study is a demonstration that older men could be changing their reasons for remaining uncircumcised, which could signal a reluctance to be circumcised despite the myriad of interventions that have been tried and showed no or minimal effectiveness.

## Supporting information

S1 File VMMC demand creation tool kit. (PDF)

S1 Data Anonymized dataset. (ZIP)
