# Supplementary material for: Shifting reasons for older men remaining uncircumcised: Findings from a pre- and post-demand creation intervention among men aged 25–39 years in western Kenya
Source: PLOS Glob Public Health. 2024 May 31;4(5):e0003188. doi: 10.1371/journal.pgph.0003188 (PMC11142559; doi:10.1371/journal.pgph.0003188)
Supplement: S1 File — (PDF) [file pgph.0003188.s001.pdf]

VMMC Demand Creation Tool Kit

Impact Research & Development Organization

IRDO Toolkit December 2014.indd 4 12/12/14 12:45 PM

VMMC

Interpersonal Communication

Toolkit for Addressing Barriers and Facilitators to Voluntary Medical Male Circumcision (VMMC) for Older Men in Nyanza Region, Kenya

IMPACT RESEARCH & DEVELOPMENT ORGANIZATION

Baring Road,
P. O. Box 9171 - 40141, Kisumu, Kenya.
Tel: +254-057-2020132; E-mail: info@impact-rdo.org (www.impact-rdo.org)

IRDO Toolkit December 2014.indd 2 12/12/14 12:45 PM

VMMC
Interpersonal Communication

Demand Creation Tool Kit

Mobilizers' Guide

Key Barriers to and Facilitators of VMMC Services

[v. 2.0, September 2014]

IMPACT RESEARCH AND DEVELOPMENT ORGANIZATION
Developing and Sustaining Empowered & Healthy Individuals & Communities

IRDO Toolkit December 2014.indd 3
12/12/14 12:45 PM

TABLE OF CONTENTS

ABBREVIATIONS AND ACRONYMS 5
ACKNOWLEDGMENTS 6
PREFACE 8
HOW TO USE THIS GUIDE 10
BARRIERS TO VMMC 12
FACILITATORS TO VMMC....................30
WOMEN'S VOICES....................40

IRDO Toolkit December 2014.indd 4 12/12/14 12:45 PM

ABBREVIATIONS AND ACRONYMS

B	-	Barrier
CDC	-	Centres for Disease Control and Prevention
DSO	-	Dedicated Service Outlets
F	-	Facilitator
GBV	-	Gender Based Violence
HIV	-	Human Immune deficiency syndrome
HPV	-	Human Papilloma Virus
IRDO	-	Impact Research and Development Organization
IPC	-	Inter Personal Communication
MC	-	Male Circumcision
STIs	-	Sexually Transmitted Infections
VMMC	-	Voluntary Medical Male Circumcision
W-B	-	Women Barrier
W-F	-	Women Facilitator

IRDO Toolkit December 2014.indd 5
12/12/14 12:45 PM

ACKNOWLEDGMENTS

This Toolkit was produced as part of a study entitled: **Male Circumcision for HIV Prevention in Kenya: Seeking Effective Strategies to Recruit Older Men.** It has been compiled with information from published literature and qualitative data (focus group discussions – FGDs, and in-depth interviews - IDIs) conducted during the formative phase of the study among men and women aged 25-39 years in the Nyanza region. The development of the Toolkit was a collaborative effort of several institutions and individuals, with contributions from VMMC experts listed below. Firstly, Julie Ambia supported by Dickens Omondi, took the lead in conducting the initial systematic review of documented barriers to and facilitators of VMMC across Africa. The rest of the team reviewed the results of the systematic review and developed the initial messages. Secondly, Jacob Onyango, Dr. Eunice Omanga and Dr. Kawango Agot reviewed the transcripts from the FGDs and IDIs and updated the Tooklit with information obtained from the same age groups as the study participants.

Special appreciation goes to the individuals representing the diverse organizations listed below:

Impact Research and Development Organization (IRDO)

- Dr. Kawango Agot, PhD, MPH
- Dr. Ohaga Spala, MSc, PhD
- Dr. Eunice Omanga, MES, DrPH
- Dr. Duncan Odera, MBChB, MPH (c)Jacob Onyango, BA, MA
- Bernard Ayieko, RCO (Dip), HND, MCHD
- Erastus Aroko, RN (Dip)
- Fidel Asol, RN (Dip), BSN (c)
- Paul Ohaga, Dip. (Journalism & Mass Communication)

Consultants:

- Dickens Omondi, HND, MPH, MSocSc, PhD (c) – Kisii University
- Julie Ambia, BSN, MPH, PhD (c) – University of Nairobi
- Richard Odindo, MSc , Communications Consultant
- Silas Achar, BA (Communications) – FHI360
- Mathews Onyango, BA, MA, MPH – FHI360

There are many others who contributed to the development of this document in one way or another, but have not been mentioned here by name. To everyone, we say '*Erokamano*'.

This Toolkit was produced and evaluated on behalf of Kenya's Ministry of Health (MOH), and was funded by the United States President's Emergency Plan for AIDS Relief (PEPFAR) through the Centers for Disease Control and Prevention (CDC) under Cooperative Agreement Number 5U01GH000518.

IRDO Toolkit December 2014.indd 6 12/12/14 12:45 PM

However, its contents are solely the responsibility of Impact Research and Development Organization (IRDO) and do not necessarily represent the official views of MOH, PEPFAR, CDC or Prevention or the Department of Health and Human Services.

IRDO Toolkit December 2014.indd 7

12/12/14 12:45 PM

PREFACE

The *Interpersonal Communication Toolkit for Addressing Barriers and Facilitators to VMMC,* is an inventory of barriers to and facilitators of the uptake of VMMC services among older men in Kenya and other parts of Africa. It is designed to serve as reference guide for VMMC mobilization, especially for men aged 25-39 years.

This reference guide is an essential tool for establishing personalized VMMC messaging to improve demand creation. Currently, VMMC mobilizers deliver the same messages about VMMC to all men regardless of age, but potential clients may have different personal barriers, concerns and questions. For example, most older men have regular partners and they may be concerned about sexual abstinence period and/or how their partners would perceive their decision to get circumcised; a large majority of older men provide for themselves and their families and have expressed concerns over lost wages during circumcision and healing period; older men are uneasy mixing with younger men and boys while waiting to be served; older men may still have concerns over breaking away from their culture of non-circumcision; older men think circumcision is for boys and younger men; and a host of other reasons. The goal of this Toolkit is to serve two purposes: i) allow the mobilizer to identify and address barriers and facilitators to VMMC uptake that are unique to each individual who has not been circumcised, and tailor the message to the specific concerns and questions raised; and ii) for each identified barrier or facilitator, the Toolkit provides talking points for the mobilizer and guides him/her on the key messages to deliver uniformly for every individual. It is hoped that this approach will help VMMC implementers improve their mobilization and subsequently increase VMMC service uptake.

The demand creation toolkit contains a range of responses and messages to be communicated to potential clients and female partners based on their particular barriers. It is organized according to barriers and facilitators to allow the mobilizer quickly refer to the relevant section of the toolkit for the appropriate responses and messages.

The toolkit development process was undertaken in two parts. First, IRDO conducted a systematic literature review of over 80 peer-reviewed articles and research studies conducted in different countries of sub-Saharan Africa on barriers and facilitators to VMMC uptake as well as on the views of female partners. About 60 relevant sources were thoroughly scrutinized and quantified in terms of how many times each barrier, facilitator and women's views were mentioned. Based on the results of the literature review, IRDO organized a two-day workshop with their core VMMC team and several external experts to review the data and to formulate responses to the barriers and messages to

IRDO Toolkit December 2014.indd 8 12/12/14 12:45 PM

reinforce the facilitators. This was followed by a formative study that provided new data about perceptions and impressions about barriers/facilitators to VMMC among circumcised and uncircumcised men aged 25-39 years in Nyanza and their partners, as well as other women. Data from the formative study were used to update the messages in the Toolkit.

Dr. Kawango Agot
Director, IRDO

HOW TO USE THIS GUIDE

Introduction:

The Communication Toolkit for Addressing Barriers and Facilitators to VMMC for Older Men in Nyanza (hereafter referred to as Toolkit) is an in-depth resource for helping older men overcome the problems/issues that are deterring them from taking up VMMC. It is designed to be used as part of a research study to assess the impact of an intervention known as Interpersonal Communication (IPC) in which men aged 25-39 years who have not gone for circumcision are approached in their homes, reasons why they have not gone for circumcision explored, and the Toolkit used to address specific barriers raised.

Research Assistants (RAs) will deliver the intervention to the participant either at enrollment into the study or if the participant is busy, the RA will make a return visit on a day and time agreed on during enrolment visit when participant is likely to be at home.

When an RA gets a potential participant in IPC study Locations, s/he will:

- Introduce self as an RA from IRDO then introduce the study using the script below:
    - Hello! My name is______________________________. I am part of a team from Impact Research and Development Organization (IRDO), Kisumu. IRDO, Centers for Disease Control and Prevention (CDC, an agency of the United States government) and the Ministry of Health are doing a research on voluntary medical male circumcision (VMMC). We would like to better understand what would make more men your age go for VMMC. This study is taking place in about 45 Locations in Nyanza Region, including this one. Sometime back we visited your village to list all males aged 25 to 39 years living in each household. During this visit, we will read a document to explain the study and ask you if you would like to take part. If you chose to take part, we will then discuss the reasons why you have not gone for circumcision.

Consenting:

- *Consent the participant if he is willing to participate in the study using Appendix 2A: Written ICF for Enrollment and Verification of MC Status.*
- *Consent each participant for verification of his MC status and, to be reached with the Intervention. Document consent on the form by ticking the appropriate box(es).*

Delivering the Intervention:

- *Strike a rapport with the participant (and his spouse if present) by making small talk for a few minutes about non-study topics. This will help put the participant at ease.*
- ***Barriers:***
    - Begin the intervention by asking the participant why he or his peers have not gone to be circumcised

IRDO Toolkit December 2014.indd 10 12/12/14 12:45 PM

    - Write down up all the reasons/responses mentioned to guide the discussion on paper provided for this purpose
- Using the relevant section of the Toolkit, address each reported barrier carefully and completely, always ensuring the person(s) is/are engaged in the discussion
    - The goal is to provide correct and complete information in a relaxed and conversational manner
- Revisit each stated barrier, exploring what they think about them after the discussion,
    - Explore whether the same barriers would still deter them/their peers from getting circumcised
- Address any new or lingering concerns.
- ***Facilitators***: After exhausting the discussion on barriers, move on the facilitators and proceed as follows:
- Strike a rapport by asking the participant if they know any benefits of VMMC
- Jot down responses to guide the discussion
- Pick each benefit/facilitator mentioned and using the relevant section of the Toolkit, reiterate the benefits,
    - Ensure sufficient and correct information is given.
- Also ask what are some of the reasons that would make them choose to get circumcised
    - Write down up all the reasons/responses mentioned to guide the discussion on paper provided for this purpose.
- Revisit each stated facilitator and explore what they think about them after the discussion,
- *Address any new or lingering concerns.*

Women's Issues:

If the spouse is present during the discussion on barriers and facilitator's, use the Women's Issues section to address her responses/concerns. Refer to the men's section if relevant.

Closing:

After addressing all the participant's/spouse's questions and concerns, to conclude;

- Encourage the men to seek VMMC and ask them to urge their family members and friends to also go for circumcision
- Leave behind a referral coupon (Appendix 15) indicating sites where VMMC is being offered in the neighborhood and contact information of team leaders in case of questions.
- If in IPC and IPC+DSO Locations, leave a copy of Appendix 17: 'All You Need to Know About VMMC' booklet with each person/family reached, for ongoing reference and to share with others.

IRDO Toolkit December 2014.indd 11 12/12/14 12:45 PM

BARRIERS TO MC

TABLE OF CONTENTS

B1. VMMC is painful 13
B2. VMMC is against my culture....................14
B3. Against Religion....................14
B4. Complications 15
B5. Procedure Cost 16
B6. Loss of Income (Lost Wages)....................16
B7. Not interested....................16
B8. Sexual Abstinence....................17
B9. Older Age....................18
B10. Lack of Knowledge of how MC Works to Prevent HIV Infection 18
B11. (a). MC Procedure (Surgery) 19
B11 (b). VMMC Procedure (Post-op) 20
B12. Healing Period 21
B13. Lack of family (partner/parent) support....................21
B14. Perceived Low HIV Risk 22
B15. Service Accessibility 22
B16. Busy/ No time....................23
B17. Married....................24
B18. Reduced Libido....................24
B19. Community Opposition....................25
B20. Exposure to HIV....................25
B21. Provider Gender 26
B22. Age Mixing 26
B23. Inadequate Partner Support....................27
B24. Reduced size of Penis 27
B25. Myths and Misconceptions 28
B26. Fear of HIV Test 28
B27. Embarrassment of Being Seen at MC Site....................28
B28. Embarrassment of Exposing Nudity 28

BARRIERS TO MC

B1. VMMC is painful

- **Explore what he has heard about the magnitude and period of pain – then say:**
  - There is slight pain during the delivery of medicine that lasts for a few seconds.
  - This occurs:
    - When injecting the medicine to prevent pain.
- Where is the injection given?
  - At the base of the penis *(show illustration)*

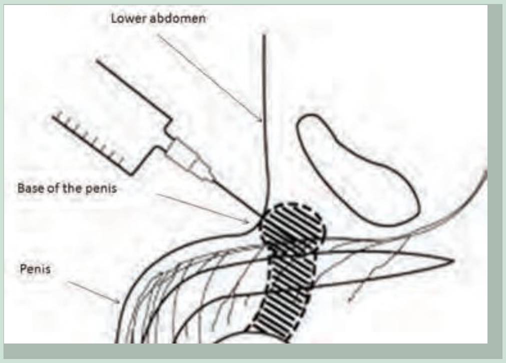

- However, most males report little or no discomfort during circumcision. After circumcision there is minimal pain, which is managed by the pain medication given, if taken as instructed.
- The slight pain will resolve within a few days after circumcision. *(Correct any additional misconceptions mentioned by the client which is not covered in the talking points above)*

Then add:

- *Most men report after being circumcised that the pain was much less than they were made to believe.*
- Remember that a large number of men your age have already been circumcised.

B1. Pain

IRDO Toolkit December 2014.indd 13
12/12/14 12:45 PM

B2. VMMC is against my culture

- **Explore what he means by culture loss, correct any misconceptions, then say:**
- VMMC is not done for cultural reasons. It is not based on any season or accompanied by any ritual. It is not done to make one identify with a community. Unlike MC for cultural reasons, VMMC is not done as a rite of passage to adulthood. Thus, being circumcised medically has nothing to do with any culture.
- VMMC means Voluntary Medical Male Circumcision; as the name suggests, this is done purely for MEDICAL reasons and is voluntary.
- The VMMC we are promoting is purely for HIV prevention & other medical benefits such as preventing certain STIs, cancer of the penis & cervix; also makes it easier to clean the genital area and to wear a condom.
- During sex men who are not circumcised can easily acquire a virus that causes cancer, known as Human Papilloma Virus (HPV).
- VMMC is done by trained health care providers while cultural MC is done by traditional circumcisers.
- Remember that a large number of men your age in this community have already been circumcised. *(Correct any additional misconceptions mentioned by the client which is not covered in the talking points above)*

B2. Against Culture/ Culture Loss

B3. Against Religion

- **Explore what client means by the concern, correct any misconceptions, then say:**
- VMMC means Voluntary Medical Male Circumcision; as the name suggests, this is done purely for MEDICAL reasons and is voluntary.
- VMMC is not done for religious reasons, for e.g., it is not accompanied by any religious ritual; it is not done to make one identify with a religion.
- What we are promoting is VMMC purely for HIV prevention & other medical benefits, such as prevention of certain STIs, cancer of the penis & cervix, and reduced acquisition/transmission of HPV, a virus that causes cancer.
- VMMC also makes it easier to clean the genital area and to wear a condom.
- VMMC is done by health care providers trained on

B3. Against Religion

IRDO Toolkit December 2014.indd 14 12/12/14 12:45 PM

VMMC and offered in a health care setting.

- Thus, it is not associated with beliefs & practices of any religion/denomination.
- In addition, there is no religion or denomination that advices its followers not to be circumcised.
- Remember that a large number of men your age across all religions in this community have already been circumcised. *(Correct any additional misconceptions mentioned by the client which is not covered in the talking points above)*

B3. Against Religion

B4. Complications

- **Explore the complications that the client is concerned about, correct any misconceptions, then say**
  - VMMC is a minor surgical procedure that is safe when done by trained health care providers.
  - However, like other surgical procedures there may be small risks such as reaction to pain medicine, swelling, infections, or accidental injury to the penis. Complications during and after surgery are rare.
  - After doing over 800,000 circumcisions in the country, less than 1% report ANY complications, most of them mild to moderate.
- While total loss of penis or death get reported with traditional circumcision, with over 800,000 procedures done in Kenya to date, no death has occurred and only two serious injuries to the penis has been reported.

Note: Explain surgical procedures if not explained earlier (see B 11 for explanation)

- It is important to seek VMMC services in a medical set up, and to follow post-operative instructions to reduce such risks.
- At the facility you will be given a hotline number to call/flash for assistance if there are any signs of complications; you can also go to the nearest health facility.
- Complications that get reported include:
  - Pain
  - Bleeding
  - Wound infection
  - Reaction to pain medicine
  - Accidental injury to the penis
  - Any other listed here which you may experience
- Remember that a large number of men your age have already been circumcised. *(Correct any additional misconceptions mentioned by the client which is not covered in the talking points above)*

B4. Complications

IRDO Toolkit December 2014.indd 15 12/12/14 12:45 PM

B5. Procedure Cost

- Explore what client means by cost as a barrier, correct misconceptions, then say:
    - Currently, VMMC is offered free of charge in government health facilities; however, a fee is charged in most private facilities.
    - All you cover is transport to the VMMC site; however, in some programs, transport arrangements are made for clients living far from the sites.
    - However, VMMC services may be provided at a cost in the future if the current funding ends.

(Correct any additional misconceptions mentioned by the client which is not covered in the talking points above)

B5. Procedure Cost

B6. Loss of Income (Lost Wages)

- Explore what client means by lost income/wages, correct misconceptions, then say:
    - Most men resume their routine work within 3 days after circumcision; those engaged in manual work such as construction workers may resume normal work within one week. Scholars have reported that participants resumed normal general duties after a day and 93% with regular employment resumed working within 1 week.
    - If your regular job is labor intensive, before you resume your normal duties, you can continue doing light duties after circumcision.
    - For the short period (i.e., three days to one week) before you resume routine work, you can save money before going for circumcision and use in buying food.
    - There is no need of special diet, care, or clothing after circumcision.
    - The health benefits from circumcision far outweigh the loss of income for the few days you may not be earning, or the cost of transport to the clinic.
    - Remember that a large number of men your age have already been circumcised.

(Correct any additional misconceptions mentioned by the client which is not covered in the talking points above)

B6. Loss of Income (Lost Wages)

B7. Not interested

- Explore (do not read) what the issues are – e.g. attitudes, fears, past experience, myths and misconceptions.
- If you identify the issues, go to the specific areas of concern,

B7. Non-Interested

IRDO Toolkit December 2014.indd 16 12/12/14 12:45 PM

- look at the constraints within the context he is in and address appropriately using relevant sections in this guide
- Look at what motivates him – the facilitators, and use appropriate sections in this Toolkit to address.

(Correct any additional misconceptions mentioned by the client which is not covered in the talking points above)

B7. Non-Interested

B8. Sexual Abstinence

- **Explore specific concerns about abstinence (also ask if period of abstinence in known), correct misconceptions, then say:**
- There are reasons why one needs to abstain for 6 weeks after circumcision:
  - It is recommended because complete healing takes 6 weeks.
- If you engage in sexual activity or masturbation before 6 weeks;
  - This may also lead to opening up of the wound or wound breakage.
  - You may increase your and your partner's risk of infection with HIV or STIs.
  - These may delay healing further and lead to more serious complications.
- Even though the wound may look like it is healed in about two weeks, note that it is not healed on the inside – this takes 6 weeks for most men.
- Continue discussing with your partner the importance of abstinence period.
- It is possible for women (and men) to abstain from sex for 6 weeks; for example, if the women can abstain for 6 weeks after delivery, then men too can abstain from sex after VMMC.
- Abstaining for 6 weeks helps the wound to heal and also prevents transmission of other STIs to their partners.
  - When you resume sex (after the 6 weeks), it is advisable to use condoms correctly and consistently.
- Remember, it is important to involve your spouse/sexual partner in decision making as you plan to seek VMMC services; this way, she will help you in abstaining.

(Correct any additional misconceptions mentioned by the client which is not covered in the talking points above)

B8. Sexual Abstinence

IRDO Toolkit December 2014.indd 17 12/12/14 12:45 PM

B9. Older Age

- **Explore why he thinks age is a factor in male circumcision; correct any misconceptions and use the appropriate section of this Toolkit to address the concerns, e.g.**
  - VMMC is beneficial across all age groups and therefore can be done at any age.
  - The healing periods as well as risks associated with VMMC are similar among adolescents and adults, so being circumcised in older age does not mean more risk or delayed healing.
  - The perception that older men who go for VMMC have or intend to have multiple partners is also not correct; studies have shown that VMMC does not promote irresponsible sexual behavior in any age group.
  - Recent studies in Kenya have shown that new HIV transmission is occurring in the older age group – those who are married or are in stable relationships. Getting circumcised is one way of reducing HIV risk in this age group.
  - Remember that a large number of men your age have already been circumcised.

(Correct any additional misconceptions mentioned by the client which is not covered in the talking points above)

B9. Older Age

B10. Lack of Knowledge of how MC Works to Prevent HIV Infection

- There are several ways that circumcision reduces the risk of HIV infection:
  - Cells that HIV attaches itself to (known as HIV target cells) are found in very high numbers in the inner foreskin – this makes it easy for the virus to get into the body through the inner foreskin.
  - These HIV target cells are located close to the surface of the skin making HIV attach easily to them; removing the foreskin during circumcision reduces the number of these cells hence reducing the risk of getting HIV during sex.
  - The surface of the inner foreskin is thin and can get bruised easily during sexual intercourse; the bruises can allow HIV to enter the body easily.
  - The fore skin is covered, moist and soft hence provides a suitable condition for germs that cause most STIs that cause wounds to live; STIs with wounds allow HIV to enter the body easily.

B10. How MC Works

IRDO Toolkit December 2014.indd 18 12/12/14 12:45 PM

- The foreskin also offers suitable condition underneath it for the virus to remain alive longer, which gives it more time to enter the body.
- After circumcision, there is hardening of the remaining portion of inner foreskin. This reduces chances of tears and wounds during sexual intercourse, which provide entry points for HIV

(Correct any additional misconceptions mentioned by the client which is not covered in the talking points above)

B10. How MC Works

B11. (a). MC Procedure (Surgery)

- **Explore what he has heard about penile injury, correct any misconceptions, then say:**
- VMMC is provided by trained health providers following the guidelines laid down by the Ministry of Health (Explain surgical procedures as follows, using sketches).

1. In the operating room, a client is examined for fitness to undergo male circumcision.
2. He is asked to lie on his back on the couch with his hands around the head
3. The genital area is then cleaned with antiseptic solution to remove any germs that may cause infection after surgery.
4. He is then covered with a green towel so as to not expose the rest of the body; the green towel has a small hole in the middle where only the penis is exposed.
5. All the instruments and supplies that are used during the procedure are new and sterile to eliminate chances of getting infection at all sites (static and mobile site).
6. The surgeon marks around the foreskin where the circumcision line will follow (2 mm from neck of the head of the penis).

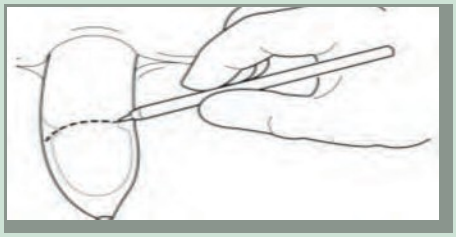

7. He is then injected with medicine at the base of his penis to prevent him from feeling pain during the surgery (show diagram under B 1).

(Demonstrate this and next 4 bullets while explaining that…)

8. The foreskin is gently pulled out and freed from the head of the penis (glans).
9. The extended foreskin is clipped beyond the glans with an

B11 (a). VMMC Procedure (Surgery)

IRDO Toolkit December 2014.indd 19 12/12/14 12:45 PM

instrument called forceps; the glans remains on one side of the forceps while the foreskin is on the other side.

10. The excess skin is then carefully and neatly cut off along the marked lines. The surgeons are very careful not to touch the glans, which is protected on the other side of the forceps.

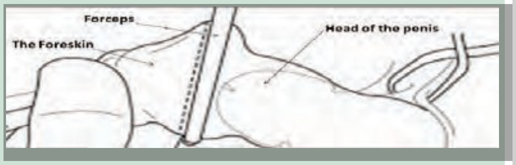

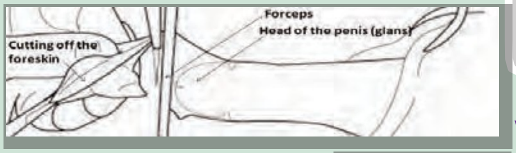

11. Bleeding vessels are identified and tied with absorbable thread, which do not require removal later.

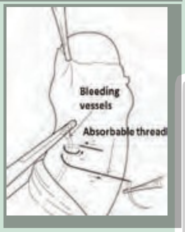

12. The wound is then closed and a dressing applied.
13. The client is given pain medicine to swallow and some to carry home, with instructions on how to take them.

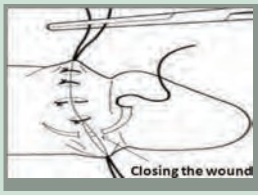

14. The client is escorted to the recovery room where he rests for 30 minutes; during this time, he is monitored for any complications before being discharged home.

- The client is given refreshment (soda) and also taught how to remove the bandage on Day 3, at home.

(Correct any additional misconceptions mentioned by the client which is not covered in the talking points above)

B11 (a). VMMC Procedure (Surgery)

B11 (b). VMMC Procedure (Post-op)

- Removal of the bandage is done by the client at home on Day 3.
- At discharge, the client is given post-circumcision instructions as follows:
    - Positioning the penis facing up to avoid swelling - clients are advised to wear tight underpants to hold the penis in the recommended position which allows clients to wear their usual trousers/shorts. Therefore, there is no need to tie a leso.
    - Removal of the dressing after 3 days.
    - How to shower or clean without contaminating the wound.
    - Need for return to the clinic after 7 days.
    - Need for 6 weeks sexual abstinence period after circumcision.

B11 (b). MC Procedure (Post-op)

IRDO Toolkit December 2014.indd 20 12/12/14 12:45 PM

- He will also be given a hotline or emergency number to call or flash any time - day or night in case anything unusual with the wound is noticed
- The healthcare providers are on standby to address any concerns.
- When these processes are followed by the surgeon and when the person being circumcised also follows the instructions given, there should be no or minimal pain or risk to the penis.
- It is important to seek circumcision in a medical setup where services are provided by trained medical staff, under safe conditions.
- It is also important to follow instructions provided by healthcare providers.

(Correct any additional misconceptions mentioned by the client which is not covered in the talking points above)

B11 (b). MC Procedure (Post-op)

B12. Healing Period

- The recommended healing period is 6 weeks if one follows the post op. instructions regardless of once age.
- Most men resume their routine work within 3 days after circumcision
- People who work sitting down resume work the same day of circumcision.
- Those engaged in manual work such us construction workers may resume normal work within one week.
  - However, you can do light tasks e.g. domestic chores or herding cattle.
- For the short period (i.e., three days to one week) before you resume routine work, you can save money before you go for circumcision and use in buying food.

(Correct any additional misconceptions mentioned by the client which is not covered in the talking points above)

B12. Healing Period

B13. Lack of family (partner/parent) support

- *Explore what partner/parent is saying against VMMC and use the appropriate section of this Toolkit to address the mentioned barriers.*

Note: *Request if you could discuss with him and his partner/parent together; if acceptable, make an appointment for a convenient time, if not the same day.*

- Explore his own barriers and facilitators and use the appropriate section of the Toolkit to address them.
- Approval by or discussion with partner/parent to reach a

B13. Lack of family (parent/ partner) support

IRDO Toolkit December 2014.indd 21 12/12/14 12:45 PM

consensus is critical before one goes for circumcision.

- The benefits of VMMC apply to spouses as well (e.g., reduce risk of cervical cancer, reduced risk of HIV and STI also benefits women, reduced acquisition/transmission of HPV. as is improved genital hygiene and ease of putting on condom)
- If indicated, refer the couple or participant and parent for counseling.

(Correct any additional misconceptions mentioned by the client which is not covered in the talking points above)

B13. Lack of family (parent/ partner) support

B14. Perceived Low HIV Risk

- **Explore reasons for perceived low HIV risk, then say:**
  - HIV can affect anyone regardless of age, marital status, profession or religion (narrow down to specific reason for his low risk perception).
  - In Kenya, new HIV transmission is occurring among men in older age groups, specifically among those who are married or in stable relationships.
  - Even for those who believe they are at no or low risk, VMMC is still beneficial because of other benefits such as reduction in risk of penile cancer to self and cervical cancer in partner, reduced acquisition/transmission of HPV, ease of maintaining genital hygiene, and ease in putting on condoms.

(Correct any additional misconceptions mentioned by the client which is not covered in the talking points above)

B14. Low HIV Risk

B15. Service Accessibility

- **Explore why he feels services are inaccessible, then say:**
  - VMMC programs strive to bring services closer to the people, mostly through mobile services within the community and outreach services in nearby health facilities.
  - VMMC is provided free of charge in all Government facilities; in some facilities the services are provided on designated days only.
  - VMMC is also provided in some private facilities, but most charge a fee.
  - However, even with these efforts, sometimes health facilities are still far in some areas.
  - Most VMMC programs are flexible and can bring services closer to you if you assemble several men who need the services or arrange local transport to take you and your

B15. Service Accessibility

IRDO Toolkit December 2014.indd 22 12/12/14 12:45 PM

friends to the nearest VMMC venue and back to your homes.

- Some VMMC programs also offer the service on convenient days and time, i.e weekends, holidays and at night (moonlight)
- Some VMMC programs will provide transportation to and from the site if the client needs to travel long distances.

(Correct any additional misconceptions mentioned by the client which is not covered in the talking points above)

- **Give a list of facilities available near the client; also provide a hotline so that he can call and be directed to the nearest service center**.

B15. Service Accessibility

B16. Busy/ No time

- **Explore what he means by being busy (or whichever equivalent term is used), address any misinformation, then discuss if:**
  - No time to go for services – discuss the schedule and advise on availability of various 'customized' services including moonlight circumcision, dedicated service outlets (if IPC or IPC/DSO), mobile services and how he can access them.
  - No time to wait for services at the clinic – let him know he can be given an appointment or information on days/time/clinics with low client flow; referral coupon can be tagged for express service at the clinic.
  - No time for sexual abstinence - (refer to Tab# B 8)
  - No time off from work - at IRDO VMMC sites you can request for up to 3 days of sick-off.
- **Remind him that going for VMMC is investing in his health and he can choose to prioritize it and create time to go for it.**
  - No time for healing period - (refer to Tab# B 12)
  - *Note: (If having no time to heal was mentioned under bullet #1&2, also say:*
    - Most men resume their routine work within 3 days after circumcision although complete wound healing takes 67 weeks; those engaged in manual work such us construction workers may resume work within one week.
    - For the short period (i.e., three days to one week) before you resume routine work, you can save money before going for circumcision and use in

B16. Busy/ No time

IRDO Toolkit December 2014.indd 23 12/12/14 12:45 PM

buying food.

- Consider that the health benefits from circumcision far outweigh the loss of income for the few days you may not be earning.
- There is no need of special diet, care, or special clothing after circumcision.

(Correct any additional misconceptions mentioned by the client which is not covered in the talking points above)

B16. Busy/ No time

B17. Married

- **Explore why he thinks being married is a barrier to getting VMMC, then discuss the following:**
  - In Kenya, there is rising infection among those in stable relationships (like marriage) – it is therefore possible that one's partner has HIV or may engage in other outside sexual relationships (mpango wa kando)
  - VMMC is also beneficial to your partner as it reduces the risk of cervical cancer in women
  - It will also make it easy to keep your genital area clean and make it easy to put on a condom – these are beneficial to you and your partner.
  - You are encouraged to involve your partner so she supports you during the healing process when sexual abstinence is recommended.
  - Thus, VMMC is beneficial to all men including those in stable, married relationships

(Correct any additional misconceptions mentioned by the client which is not covered in the talking points above)

B17. Married

B18. Reduced Libido

- There is no consistent evidence of decrease (or increase) in sexual performance in men or sexual satisfaction in either men or women.
- VMMC has no link with sexual performance as this depends on individual's own perception, circumstances, state of physical and mental health, and several other factors.
- Going for VMMC services may provide an opportunity where other health needs may be identified and addressed or appropriately referred.

(Correct any additional misconceptions mentioned by the client which is not covered in the talking points above)

B18. Reduced Libido

IRDO Toolkit December 2014.indd 24 12/12/14 12:45 PM

B19. Community Opposition

- **Explore what community opposition client is referring to, then discuss:**
  - Most communities that do not traditionally perform male circumcision do not teach against it either; it just does not form one of their marks of identity.
  - What many non-circumcising communities oppose is traditional circumcision – specifically those performed by traditional circumcisers or during designated seasons or in designated locations such as valleys, rivers, forests or hills/mountains;
    - These are mostly done as a rite of passage from childhood to adulthood and accompanied by rituals.
  - On the contrary, what we are promoting is Voluntary Medical Male Circumcision for HIV prevention and other medical benefits for you as an individual.
  - Some of the benefits of VMMC are reduction in the risk of certain STIs, cancer of the penis and cervix, reduced acquisition/transmission of HPV; VMMC also makes it easier to clean the genital area and to put on a condom.
- Remind him that VMMC is done by health care providers trained on VMMC, and performed in medical settings hence different from traditional circumcision.
- Reiterate that the decision to be circumcised solely lies with him that is why it is voluntary.
- Remind him that getting circumcised or remaining uncircumcised should be a personal decision and not influenced by his community.
- Remind him also that a large number of men your age in this community have already been circumcised.

(Correct any additional misconceptions mentioned by the client which is not covered in the talking points above)

Note: If it emerges that a community member (or the spouse) is influencing client's decision, seek permission to meet both and address concerns from this person as well)

B19. Community Opposition

B20. Exposure to HIV

- **Explore how in his opinion MC exposes one to HIV, correct any misconceptions (e.g. shared instruments – blades, etc), then say:**
  - VMMC is medical procedure provided by trained health care providers under hygienic conditions.
  - The instruments used are sterilized/decontaminated while gloves, blades and needles are new and not

B20. Exposure to HIV

IRDO Toolkit December 2014.indd 25 12/12/14 12:45 PM

shared; they are used and discarded after use on each client.
- However, infection could be possible in other settings where hygienic conditions are not maintained, such as during traditional circumcision.

- **Encourage him to seek VMMC services in a health care setting.**

(Correct any additional misconceptions mentioned by the client which is not covered in the talking points above)

B20. Exposure to HIV

B21. Provider Gender

- **Inquire about what the concerns are, correct any misinformation, then say:**
  - Both male and female staff provide VMMC at most sites – either as surgeons, assistant surgeons, or counselors.
  - Those performing the surgery are medically trained and also trained on VMMC; they include Medical Officers, Clinical Officers and Nursing Officers.
  - In terms of quality, both genders provide high quality services due to the standard training they undergo. They also use standard VMMC guidelines, and are supervised by senior staff from the program implementers, donors and Ministry of Health.
- In terms of concerns over being served by a female provider due to cultural or personal reasons, inform him that he may be free to choose who to perform the VMMC on him where adequate staff are available; where this choice is not available he can be referred to nearby sites where there are male-only service providers (give examples of such in the neighborhood). *(Correct any additional misconceptions mentioned by the client which is not covered in the talking points above)*

B21. Provider Gender

B22. Age Mixing

- **Find out what issues he has with age mixing, correct any misinformation, then:**
  - Explore this perception of young and old and what he recommends should be done without locking any age group out.
  - Inform him that while age mixing is common in most VMMC sites, we have sites where mostly older men come for services at designated times i.e. moonlight services, during school days (mention where sites are; in IPC and IPC/DSO sites, give direction to nearby facilities).

(Correct any additional misconceptions mentioned by the client which is not covered in the talking points above)

B22. Age Mixing

B23. Inadequate Partner Support

- Explore what the female partner is saying against VMMC and use the appropriate section of the Toolkit to address the barriers
- Request if you could discuss VMMC with him and his partner together; if necessary make an appointment for a convenient time
- Inform her/the couple that a large number of men have already been circumcised and their partners are happy with the service.
- Mention that VMMC is also beneficial to women as it reduces the risk of cervical cancer, and provides indirect protection against HIV and STIs.
- Cite that partner involvement is also encouraged to support sexual abstinence during healing.
- Add that lack of adequate knowledge may hinder women participation in VMMC services
- Explain to them the importance of the education and messages given during counseling and how it benefits both of them.
- Also explain that VMMC is not traditional male circumcision; it is medical with no rituals, and women are allowed to be involved in the process.
- Explain that women do have a responsibility for their own and their partners' health, and supporting VMMC is one such gesture of responsibility.
- In summary, women's involvement is important to enhance:
    - advocacy for VMMC,
    - support partner during healing period,
    - knowledge on the benefits of VMMC to partner and self when the partner is circumcised,
    - support for children ≥10 years to go for circumcision

(Correct any additional misconceptions mentioned by the client which is not covered in the talking points above)

B23. Inadequate Partner Support

B 24. Reduced size of penis

- MC involves the removal of the foreskin only and does not affect the length of the penis.
- MC surgery involves the removal the excess skin that the surgeon carefully and neatly cut off along the marked lines (refer to Tab # B11)
- The surgeons are very careful not to touch the glans, which is protected on the other side of the forceps (refer to Tab # B11).

B 24. Reduced size of penis

IRDO Toolkit December 2014.indd 27 12/12/14 12:45 PM

B 25. Myths and misconception

- Disposal of foreskin:
  - VMMC centres across the country operated under strict conditions which compelled them to burn foreskins soon after conducting circumcision.
  - The foreskins together with other medical wastes are put in disposal bins before being taken for incineration together with other medical waste so as not to cause harm to anybody.
  - There is no way that foreskins can find their way out of the male circumcision centres to be used for other purposes.
- Reduced libido and sexual performance (refer to Tab # B18 and W.B3)

B 25. Myths and misconception

B 26. Fear of HIV test

- It is recommended that you are tested for HIV before being circumcised and know your status. However, it is not mandatory.

B 26. Fear of HIV test

B 27. Embarrassment of being seen at MC site

- Some VMMC programs offer the service on convenient days and time (i.e weekends, holidays, at night-moonlight). You can choose to go for circumcision on a day and/or time that convenient to you.
- You can also choose to go for circumcision at a site where you are comfortable

B 27. Embarrassment of being seen at MC site

B 28. Embarrassment of exposing nudity

- During the MC procedure, a client is required to remove his clothes and put on theater gown.
- However, during the procedure, it is only the penis that will remain exposed to allow the surgeon to operate on the tip.
- During the procedure, the operation couch is screened-off to create privacy.

B 28. Embarrassment of exposing nudity

IRDO Toolkit December 2014.indd 28 12/12/14 12:45 PM

BARRIERS TO MC NOTES

IRDO Toolkit December 2014.indd 29
12/12/14 12:45 PM

FACILITATORS TO VMMC

TABLE OF CONTENTS

F1. HIV/STIs Protection....31
F2. Health Benefits 32
F3. Sexual Pleasure/ Performance....33
F4. Procedure Safe....33
F5. Family/Community Support....33
F6. Culturally Acceptable....34
F7. Religious Support 34
F8. No Increase in Risk Behaviors 35
F9. Peer pressure/ Social Acceptability....36
F10. VMMC is a Short Procedure 36
F11. Recommended....36
F12. Positive Staff Attitude....37
F13. Pain is Minimal....37
F14. Real Man....37
F15. Stakeholder Support....36
F16. "Incentives" 38
F17. Cultural Identity & Practice....38
F18. VMMC Free Services 38
F 19. Penile appearance 39

FACILITATORS TO VMMC

Messages

F1. HIV/STIs Protection

- **Probe what the person knows about VMMC protection against HIV, then correct myths & misconceptions.**
- **If not mentioned, ensure the following are covered:**
  - Three studies conducted and all found that VMMC is protective against HIV acquisition in men by about 60%
  - Being circumcised protects against HIV through the following mechanisms:
    - Cells that HIV attaches itself to (known as HIV target cells) are found in very high numbers in the inner foreskin – this makes it easy for the virus to get into the body through the inner foreskin.
    - These HIV target cells are located close to the surface of the skin making HIV attach easily to them; removing the foreskin during circumcision reduces the number of these cells hence reducing the risk of getting HIV during sex.
    - The surface of the inner foreskin is thin and can get bruised easily during sexual intercourse; the bruises can allow HIV to enter the body easily.
    - The fore skin is covered, moist and soft hence provides a suitable condition for germs that cause most STIs that cause wounds to live; STIs with wounds allow HIV to enter the body easily.
    - The foreskin also offers suitable condition underneath it for the virus to remain alive longer, which gives it more time to enter the body.
    - After circumcision, there is hardening of the remaining portion of inner foreskin. This reduces chances of tears and wounds during sexual intercourse, which provide entry points for HIV.
  - The protection is not full or 100%; this means that if you have unprotected sex with a HIV-infected partner or one whose HIV status you do not know, you may still get infected. As such, use of condoms and knowing one's HIV status is still encouraged even when one is circumcised.
- ***If STI prevention is mentioned as a facilitator:*** **Explore why he feels that prevention/ treatment of STI is a facilitator**

F1. HIV/STIs Protection

FACILITATORS TO MC

and correct any misconception.

- **Refer to the relevant section of the tool kit:**
- STI screening & treatment (See F2)
- STI prevention (See F2)
- Inform him that VMMC provides an opportunity to access other male sexual and reproductive services e.g. free STI screening and treatment

F1. HIV/STIs Protection

F2. Health Benefits

- **Explore which illnesses they know are protected by VMMC and correct any misconceptions and ensure the following are covered;**
  - VMMC reduces the risk of certain STIs, specifically those that cause wounds. These STIs include Syphilis and other STIs that present with wounds like Genital Ulcer Diseases, Genital Warts, Genital Herpes and Chancroids.
  - VMMC also reduces the risk of HIV acquisition among men by approximately 60%.
  - During sex, men who are not circumcised can easily acquire a virus that causes cancer, known as Human Papilloma Virus (HPV). Men can transfer this virus to their partners during sex. With repeated exposure to this virus, women may develop cervical cancer. Circumcision reduces the chance of acquiring HPV.
  - HPV also causes cancer of the penis, and VMMC protects men from acquiring HPV hence reducing the risk of penile cancer.
  - VMMC improves genital hygiene;
    - VMMC makes the area under the foreskin easier to clean.
    - Good penile hygiene also reduces irritation of the head of the penis.
    - It reduces bad smell from under the foreskin
  - People have also reported that putting on a condom is easier after VMMC.
- Emphasize that VMMC is not a natural condom therefore, even after VMMC, there is need to continue using condoms correctly and consistently for effective HIV prevention.
  - Medical benefits of VMMC include prevention of difficulties in retracting the foreskin in men who have conditions where the opening of the foreskins is tight.

F2. Health Benefits

IRDO Toolkit December 2014.indd 32 12/12/14 12:45 PM

F3. Sexual Pleasure/ Performance

- There is no consistent scientific evidence that VMMC enhances sexual pleasure or performance.
- Some individuals have reported enhanced sexual pleasure after VMMC while others have said there is no difference in sexual pleasure or performance before and after circumcision.
- Uncircumcised men are prone to tears during sex; this might make sex painful and less pleasurable.
  - When such pain reduces after circumcision, they may think sex is pleasurable because of circumcision
- Sexual pleasure is influenced by many other factors e.g. state of the physical and mental mind, preparedness, pressure of life, individuals own perception, general health status, etc.
- VMMC is provided for prevention of HIV and other STIs, and for other health benefits; it is not provided to enhance sexual pleasure or performance.

F3. Sexual Pleasure/ Performance

F4. Procedure Safe

- **Yes.** VMMC is safe since it is done by trained healthcare providers in hygienic conditions.
- Like any other surgical procedures, there are unexpected problems or complications that may occur, such as reaction to pain medicine, swelling, infection or accidental injury to penis. However, these are extremely rare.
- In case of any problem, VMMC programs have 24 hrs emergency response services.
- The VMMC program is offered under hygienic environment that is specifically prepared to ensure that the procedure is safe.
- The instruments used are sterilized/uncontaminated while gloves, blades and needles are discarded after use and not reused.
  - As such, both equipment and supplies that are used are either new or disinfected, as appropriate.

F4. Procedure Safe

F5. Family/Community Support

- While family support is important, the final decision to be circumcised should mostly lie with you and your partner.
- VMMC programs continuously engage community and other leaders to promote service uptake. Working together with these leaders is an ongoing process.
- VMMC is offered for HIV prevention and other medical

F5. Family/ Community Support

IRDO Toolkit December 2014.indd 33 12/12/14 12:45 PM

benefits such as preventing certain STIs, cancer of the penis and cervix, reduced acquisition/transmission of HPV; also makes it easier to clean the genital area.
- VMMC is done by health care providers trained on VMMC.
- Discussing with your partner before going for circumcision is highly encouraged

F5. Family/ Community Support

F6. Culturally Acceptable
- **Explore why they think VMMC is culturally acceptable**
- **Identify any myths or misconceptions and correct using relevant sections of this Toolkit**
- **Emphasize that VMMC is promoted for medical and not cultural reasons (see B 2);**
  - Although VMMC is being promoted for medical reasons, every effort has been made to obtain support from political, religious and other leaders at community and national levels. Working together with these leaders is an ongoing process.
  - While MC is performed among many African communities as a rite of passage from boyhood to adulthood, VMMC which we are promoting is not offered for this reason; instead, VMMC is offered because of its medical benefits such as reduction in HIV and other STIs, reduced acquisition/transmission of HPV, making cleaning of genital area easy, reducing the chance of getting cancer in the man's penis and the partner's cervix, among other benefits.
  - Those who undergo circumcision as a rite of passage also reap the same health benefits; however, any circumcision is not 100% protective against HIV and other STIs, thus abstinence or safer sexual practices is still encouraged.

Note: In the discussion, do not portray VMMC as being promoted for cultural reasons

F6. Culturally Acceptable

F7. Religious Support
- **Explore why they think VMMC is practiced for religious reasons**
- **Identify any myths or misconceptions and correct using relevant sections of this Toolkit**
- **Emphasize that VMMC is promoted for medical and not religious reasons (see B 3);**
  - While MC is performed among Muslims and some Christian denominations for religious purposes, VMMC that we are promoting is not offered for religious reasons

F7. Religious Support

IRDO Toolkit December 2014.indd 34 12/12/14 12:45 PM

but for its medical benefits, which include: reduction in HIV and other STIs, reduced acquisition/transmission of HPV, making cleaning of genital area easy, reducing the chance of getting cancer in the man's penis and the partner's cervix, among other benefits.

- Even though Muslims and others are circumcised for religious reasons, they also reap the same health benefits; however, any circumcision is not 100% protective against HIV and other STIs and abstinence, knowing your status or safe sex are still encouraged.
- Although VMMC is being promoted for medical reasons, every effort has been made to obtain support from religious and other leaders at community and national levels. Working together with these leaders is an ongoing process.

Note: In the discussion, do not portray VMMC as being promoted for religious reasons

F7. Religious Support

F8. No Increase in Risk Behaviors *(i.e. ok to be circumcised and remain with 'normal' libido)*

- **Explore why they feel that engaging in risky sexual behavior is a facilitator and correct any misconception**
- **Reinforce that VMMC is protective against HIV and other STIs but the protection is partial (60%); thus emphasize the need for ABC (Abstinence, Being Faithful, Condom use) and the importance of knowing one's own HIV status**
- **Acknowledge that having one or more casual sex partners puts him at higher risk of acquiring HIV infection and other STI infections and explain to him that:**
    - Many studies have shown that people who go for VMMC do not engage in risky sexual behaviors; for instance, they do not add more sexual partners and do not stop using condoms if they were doing so previously.
    - This means they are aware that being circumcised does not mean one is fully protected against HIV – advice the person that even if circumcised, he is not fully protected against HIV and should continue to abstain from sex, know partner's status or practice safe sex.
    - During VMMC, clients are educated and counseled on HIV risk reduction and offered HTC services (though testing is optional), including condom promotion and distribution.

F8. No Increase in Risk Behaviors

IRDO Toolkit December 2014.indd 35 12/12/14 12:45 PM

FACILITATORS TO MC

F9. Peer pressure/ Social Acceptability

- Explore what the peers are saying about VMMC or doing after VMMC and use the appropriate section of this Toolkit to correct myths or misconceptions and enhance the facilitators
- Explore their own knowledge on VMMC and use the Toolkit to fill the gaps if any, including risks and benefits.
- Encourage the person to make individual decisions that affect his own health; emphasize that the decision to go for VMMC should personal even if the views of peers are important.
- If issue is social acceptability, explore what they mean and correct any misperceptions, and tell him that;
    - In communities or among religions where MC is the norm, VMMC may make one fit into the social network.
    - However, the VMMC being promoted is for HIV prevention and other health benefits and not to make one fit into any social group.
    - If one gets accepted by peers or social networks after being circumcised, that is an added advantage but not the reason VMMC is being promoted.

F9. Peer pressure/ Social Acceptability

F10. VMMC is a Short Procedure

- **Yes.** VMMC is a minor surgical procedure done by trained and competent healthcare providers.
- The surgical procedure takes about 15 minutes; in addition, there are other associated procedures such as counseling, medical check-up, resting after surgery that takes about 30 minutes.
- VMMC does not require hospitalization and you can resume normal duties within 3 days; it takes about 5 days to resume manual work.
    - However, you can start doing light domestic work immediately after circumcision, such as cleaning the compound or washing clothes.

F10. VMMC is a Short Procedure

F11. Recommended

- In case they mention having been informed about VMMC by someone else, explore what information they received (barriers and facilitators) and use appropriate section in this document to correct where necessary.

F11. Recommended

F12. Positive Staff Attitude

- Yes, health care workers are trained on good personal relations with patients they serve, and there is strong supervision that ensures this good relationship is maintained.
- There are suggestion boxes where clients put their views; senior staff collect these views and select those they can implement to improve the services based on the suggestions.

F12. Positive Staff Attitude

F13. Pain is Minimal

- There's slight pain that lasts for a few seconds during injection of the pain medicine.
- However, during circumcision, there is no pain.
- After circumcision, you are given pain killers to take while at home; if you follow instructions, there will be minimal pain or discomfort that may last for a few days.

F13. Pain is Minimal

F14. Real Man

- Explore why he feels VMMC makes people real men, correct any misconceptions identified using the relevant parts of the Toolkit. Tell him that;
  - Some clients report this, but VMMC is promoted for medical reasons and not as a rite of passage to adulthood or a practice that makes one become a real man.
- If the issue is "enhancement of masculinity" explore what they mean and correct any myths and misconceptions, e.g. sexual performance (see B18 & F3), chauvinistic attitude, etc.
- Explain that there is no scientific link between VMMC and enhancement of masculinity.

F14. Real Man

F15. Stakeholder Support

- Although the circumcision being promoted is medical, every effort has been made to obtain support from political, religious and other leaders at community and national levels. Working together with these leaders is an ongoing process.
- Emphasize to the participants that although we appreciate the support from the political and religious leaders, the decision to be circumcised should remain with an individual and his partner because of the health benefits.

F15. Stakeholder Support

IRDO Toolkit December 2014.indd 37 12/12/14 12:45 PM

F16. "Incentives"

- **Explore what they call "incentives" and correct misinformation**
- **Explain why some programs give these items during the procedures (e.g. sodas, underwear, transport);**
    - Soda – is given to re-energize clients since they sometimes spend long hours at the clinic when many people have turned up for the services; some people also come for VMMC on empty stomachs and may react to the pain medicine.
    - Underwear – is provided to those with inadequate types, to help position the penis and thus reduce swelling and enhance healing.
    - Transport - in very rare cases, transport arrangements may be provided to clients who come for VMMC services far away from their homes.
- Explain other services that may attract clients to seek VMMC, e.g. STIs screening & treatment and other sexual health problems.

F16. "Incentives"

F17. Cultural Identity & Practice

- **Explore how they perceive VMMC as a cultural practice and note that:**
    - While some people go for MC as a cultural practice, VMMC that we are promoting is not offered for this reason; instead, VMMC is offered because of its medical benefits such as reduction in HIV and other STIs, reduced acquisition/transmission of HPV, making cleaning of genital area easy, reducing the chance of getting cancer in the man's penis and the partner's cervix, among other benefits.
    - VMMC is thus a medical procedure done by trained health care personnel in a health care setting.
    - If what attracts you to VMMC is cultural identity, that is just an added advantage.

F17. Cultural Identity & Practice

F18. VMMC Free Services

- **Yes.** VMMC is currently being provided free of charge to clients in all government facilities.
- VMMC is also provided in some private and mission health facilities but at a fee.
- Give a list of government facilities available near the client

F18. VMMC Free Services

IRDO Toolkit December 2014.indd 38 12/12/14 12:45 PM

F 19. Penile appearance

- Some circumcised men report that the circumcision improves the shape/appearance of the penis.
- However, VMMC that we are promoting is not offered for this reason; instead, VMMC is offered because of its medical benefits such as reduction in HIV and other STIs, reduced acquisition/transmission of HPV, making cleaning of genital area easy, reducing the chance of getting cancer in the man's penis and the partner's cervix, among other benefits.

F 19. Penile appearance

FACILITATORS TO MC

FACILITATORS TO MC NOTES

IRDO Toolkit December 2014.indd 39 12/12/14 12:45 PM

WOMEN'S VOICES

TABLE OF CONTENTS

W-B1. Fear of Promiscuity by Male Partners 41
W-B2. Gender Based Violence (GBV) 42
W-B3. Reduction in Libido...42
W-B4. Couple Faithful 42

WOMEN'S VOICES

Barriers

Messages

W-B1. Fear of Promiscuity by Male Partners *(Risk Compensation)*

- **Explore why she thinks VMMC leads to promiscuity (among male sexual partners) and address any mis-information using appropriate sections of this Toolkit (See F8). Explore exact concerns, addressing misinformation (e.g., VMMC improves sexual performance, increases libido, etc) with relevant sections of the Toolkit. Mention that:**
  - Results from several studies have shown that men who are circumcised do not engage in riskier sexual behaviors compared to men who have remained uncircumcised.
  - As such, there is no evidence that circumcising men would make them more or less promiscuous.
  - As a partner, women should encourage their partners to be circumcised and support them before, during and after the process; importantly, they need to go for counseling and testing together, where they will also be educated on VMMC together.
  - Emphasize that partners should discuss, ahead of going for VMMC, how to abstain from sex during the 6 weeks healing period. This discussion should include assurance that both will remain faithful to each other during and after this period.
  - During VMMC, men are informed that circumcision is partially protective against acquisition of HIV, by 60%; circumcised HIV negative men can still acquire HIV from their HIV infected partners and circumcised HIV positive men can transmit the virus to their HIV uninfected partners.
  - Thus, other known HIV prevention methods should be used even after circumcision e.g. abstinence, being faithful, condom use etc.
- **Explain the importance of going for VMMC together - they will be given education on VMMC and other HIV prevention methods,**
  - The woman will also be empowered and can remind the man about the information given during counseling.

W-B 1. Fear of Promiscuity

IRDO Toolkit December 2014.indd 41 12/12/14 12:45 PM

W-B2. Gender Based Violence (GBV)

- Explore why she believes VMMC leads to GBV, with examples if available; discuss ways to minimize GBV:
  - Discuss VMMC together as partners before the procedure
  - Seek VMMC education and counseling as a couple
- Opt to be tested for HIV together before the man is circumcised; if your partner is tested alone and the test is positive, he will be advised on how to disclose to you, or the Counselor who tested him will refer the two of you where to get necessary services.

W-B2. GBV

W-B3. Reduction in Libido

- **Explore what the client means by reduction in libido and address misconceptions**
- **Explain that results are not consistent about the effect of MC on sexual function - men in most studies have reported no difference in sexual experience before circumcision compared to after circumcision while a few have reported some improvement.**
  - However, men have not reported a reduction in sexual function.
  - As a partner, women should encourage their partners to be circumcised and support them before, during and after the process; importantly, they need to go for counseling and testing together, and also be educated on VMMC together.
- Stress that partners should discuss, before the man goes for VMMC, how to abstain from sex during the 6 weeks healing period. This discussion should include assurance that both will remain faithful to each other during and after this period.

W-B3. Change in Libido

W-B4. Couple Faithful

- **Explore why s/he feel that if a couple is faithful then the man should not be circumcised, correct any misconceptions and explain to him that;**
  - Other benefits of VMMC include prevention of penile and cervical cancer to female sex partners, reduced transmission of HPV to female partners, improved genital hygiene which is also beneficial to the woman whether she believes they are both faithful or not.
  - MC is also used as an entry point to other sexual and

W-B 4. Couple Faithful

reproductive health for men, and this may be beneficial to women too.

- Explain that research has shown that majority of HIV infections occur in stable relationships where couples are assumed to be faithful, such as in marriages; thus marriage does not necessarily offer protection against HIV to both women and men.

W-B4. Couple Faithful

WOMEN'S VOICES NOTES

....

....

....

....

....

....

....

....

....

....

....

....

....

....

....

....

IRDO Toolkit December 2014.indd 43
12/12/14 12:45 PM

FACILITATORS

TABLE OF CONTENTS

W-F1. Partner Support 45

FACILITATORS

FACILITATORS

Message

W-F1. Partner Support

- Explore type of partner support and appreciate the same. Note the following;
  - VMMC is also beneficial to women as it reduces the risk of developing cervical cancer.
  - Partner involvement is encouraged to offer support, especially in observing sexual abstinence during the healing period
- Reinforce other benefits of VMMC to women e.g. reduced risk of HIV, STI and HPV among men mean less risk to their partners; circumcised men find it easier to clean their genital area and this benefits women too; men find it easier to use condoms after circumcision; VMMC offers an opportunity for partners to be educated together and tested for HIV together; when men got for VMMC, they have an opportunity to ask the health provider other questions about their sexual and reproductive health.
- Encourage partner involvement in decision about VMMC and say;
  - Most women support their partners' decision to become circumcised if they are informed before the man goes for the procedure.
  - There are reports that when the man discusses with his partner his intention to go for VMMC, the two plan on how to observe the period of post-operative sexual abstinence.
  - Some women report that when their partners are circumcised, they are less worried about acquiring STIs including HIV, from him.
  - When you resume sex after six weeks post-circumcision, it is advisable to use condoms correctly and consistently – this is because VMMC is not 100% protective.

W-F1. Partner Support

IRDO Toolkit December 2014.indd 45 12/12/14 12:45 PM

IRDO Toolkit December 2014.indd 2
